# BMC Biochemistry Reviewer Acknowledgement, 2013

**DOI:** 10.1186/1471-2091-15-1

**Published:** 2014-01-18

**Authors:** Thomas A Rowles

**Affiliations:** 1 BioMed Central, Floor 6, 236 Gray’s Inn Road, London WC1X 8HB, UK

## Abstract

**Contributing reviewers:**

The editors of BMC Biochemistry would like to thank all our reviewers who have contributed their time to the journal in Volume 14 (2013).

## 

Niels Agerbirk

Denmark

Christophe Ampe

Belgium

Panagiotis Arapitsas

Italy

Jose Bayascas

Spain

Alexander Baykov

Russian Federation

Aaron Beedle

USA

Muhammad Bhanger

Pakistan

Haq Nawaz Bhatti

Pakistan

Ricardo Biondi

Germany

Christina Bohlin

Sweden

Nanette Boyle

USA

Isabelle Breloy

Germany

Tracy Brooks

USA

Valentina Calamia

Spain

Francisco Campos

Brazil

Beatriz Castilho

Brazil

Matteo Castronovo

Italy

Chung-Soon Chang

South Korea

Suresh Kumar Chitta

India

Roland Chiu

Netherlands

Benjamin Clemens

USA

Timothy Cole

Australia

Jane Elizabeth Collins

United Kingdom

Lynne Coluccio

USA

James Cowan

USA

Julia Cricco

Argentina

Elisabeth Daub

Canada

Henrik Hjarvard De Fine Licht

Denmark

Joris Delanghe

Belgium

Jeremy Derrick

United Kingdom

Arunkumar Dhayalan

India

Jiajie Diao

USA

Vernon Dolinsky

Canada

Lynn Dustin

USA

Ragnhild Eskeland

Norway

Gennaro Esposito

Italy

Jed Fahey

USA

Janet Finer-Moore

USA

Nick Fisk

USA

Octavio Franco

Brazil

Zheng Fu

USA

Junping Gao

China

Scott Garman

USA

Takuya Genda

Japan

Peter Glazer

USA

Meir Goldsmith

Israel

Hervé Guillou

France

Lee Hagey

USA

Jianghui Hou

USA

Wenbing Hu

USA

Cheng-Yang Huang

Taiwan

Reuben Huber

Canada

Jon Huibregtse

USA

Ibtessam Hussein

Saudi Arabia

Darrell Irvine

USA

Takao Iwawaki

Japan

Stefan Janecek

Slovakia

Caroline Jefferies

Ireland

Yuhua Ji

China

Tom Jovin

Germany

Renata Jurkowska

Germany

Erdem Karatekin

USA

David Kelly

United Kingdom

Petri Kursula

Finland

Hideo Kusaoke

Japan

Robert Lahue

Ireland

Dalit Landesman-Milo

Israel

Giovanna Lattanzi

Italy

Enzo Laurenti

Italy

Pedro A. Lazo

Spain

Seng-Kee Leong

Australia

Christina Leslie

USA

Sanford Leuba

USA

Randolph Lewis

USA

Matthew Lord

USA

Van Luu-The

Canada

Victor Luzhkov

Russian Federation

Ross Macgillivray

Canada

Serge Manie

France

Sandra Marmiroli

Italy

Takeshi Matsumura

Japan

James Mcnew

USA

Miriam Mecha

Spain

Amy Medlock

USA

Gustavo Menezes

Brazil

Shigeki Miyamoto

USA

Michel Moenner

France

Luca Morini

Italy

Iouri Motorine

France

Bishnu Mukhopadhyay

India

Ernesto Nakayasu

USA

Senthil Natesan

Honduras

Predrag Novak

Croatia

Maria Manuel Oliveira

Portugal

Michael Palmer

Canada

Carrie Partch

USA

John Patterson

Afghanistan

Danilo Porro

Italy

Pradman Qasba

USA

Hermann Ragg

Germany

Emma Raven

United Kingdom

Thore Rohwerder

Germany

David Rose

Canada

Frank Sargent

United Kingdom

Thomas Scheibel

Germany

Jon Seal

USA

John Sedbrook

USA

Halyna Semchyshyn

Ukraine

Dihua Shangguan

China

Ihsan Shehadi

United Arab Emirates

Keiro Shirotani

Japan

Stefan Siemann

Canada

Mara Silveira Benfato

Brazil

Wipa Suginta

Thailand

Tomasz Szkudelski

Poland

Yuko Tsutsui

USA

Julio A. Urbina

Venezuela

Vladimir Uversky

USA

Claudio Varotto

Italy

Pablo Velasco

Spain

Miguel Vidal

Spain

Alexander Volkov

Belgium

Fumio Watanabe

Japan

John Whitfield

Australia

Curtis Wilkerson

USA

Elizabeth Wilson

USA

Duane Winkler

USA

Yongmei Xia

China

Dimitris Xirodimas

France

Changcheng Xu

USA

Huaxi Xu

USA

Kaitian Xu

China

Tsong-Rong Yan

Taiwan

Yuichi Yoshimura

Japan

Hiroya Yurimoto

Japan

Snezana Zaric

Serbia

Yanqiao Zhang

USA

Xinqing Zhao

China

Lei Zhou

USA

Danna Zimmer

USA

